# Winged Fruits of *Deviacer* in the Oligocene from the Ningming Basin in Guangxi, South China

**DOI:** 10.1371/journal.pone.0144009

**Published:** 2015-12-01

**Authors:** Yunfa Chen, Steven R. Manchester

**Affiliations:** 1 Guangxi Museum of Natural History, Nanning, Guangxi, 530012, China; 2 Florida Museum of Natural History, University of Florida, Gainesville, Florida, 32611-7800, United States of America; Institute of Botany, CHINA

## Abstract

*Deviacer guangxiensis* Chen & Manchester sp. nov. is described based on asymmetric samaras from the Oligocene Ningming Formation in Guangxi, South China, representing the first documentation of *Deviacer* fossils in Asia. The Oligocene species, with relatively large fruits, represents the youngest record of the genus so far known; all other records are from the Paleocene and Eocene, or late Eocene—early Oligocene in western North America and Europe. It indicates that the extinct genus, *Deviacer*, was widely distributed in the northern hemisphere during the Paleogene.

## Introduction

A variety of winged fruits are preserved along with fossil leaves in Oligocene lacustrine deposits of the Ningming Formation in Guangxi Zhuang Autonomous Region, South China. In addition to those of Juglandaceae [1,2], other winged fruits include *Ace*r L., *Ailanthus* Desf., *Chaneya* Wang & Manchester, *Fraxinus* L., and others. Here we describe a new species of the extinct genus, *Deviacer* Manchester.

Perhaps the most common type of asymmetric samara is the kind exemplified by the genus *Acer*. This kind of wind-dispersed fruit, having a single elliptical seed and an extended lateral wing with numerous subparallel and distally arching veins, also occurs in various angiosperm families including Anacardiaceae (*Loxopterigium*), Leguminosae (*Tipuana*), Malpighiaceae (*Banisteria*), Malvaceae (*Tarrietia*, *Triplochiton*), Phytolacaceae (*Gallesia*, *Seguieria*), Sapindaceae (*Diatenopteryx*, *Thinouia*), Ulmaceae (*Phyllostylon*), and Polygalaceae (*Securidaca*). Many of these were reviewed by Mirle and Burnham [3,4]. Evidently, this fruit type has evolved multiple times in different groups of Rosids. The extinct genus *Deviacer*, previously documented only from the Paleocene and Eocene, or late Eocene—early Oligocene in western North America, is an example [5,6].

The fossil fruits informally assigned to “*Acer arcticum*” from North American [4] had been noted to differ from typical *Acer* winged fruits in having a dorsally, rather than ventrally, directed wing. It was suggested that these fossil fruits might represent an extinct genus of Aceraceae by Wolfe and Tanai [4]. The compressed fruits of the “*Acer arcticum*” were later formally described as an extinct genus, *Deviacer*, and assigned to Sapindaceae sensu lato [5]. Based on material from the late Eocene—early Oligocene Badger’s Nose flora of northern California, the genus is emended and attributed to the family Polygalaceae [6]. These authors considered that the morphological differences of *Deviacer* from extant *Securidaca* are minor and insignificant and therefore assigned the fossil genus to the same family, However, the similarities between *Deviacer* and *Securidaca* may be result of convergent evolution, thus we prefer to leave the familial assignment of *Deviacer* open, although we agree that it likely represents a Rosid Eudicot.

*Deviacer* and *Acer* differ fundamentally in mode of attachment. *Acer* fruits are schizocarpic, with a pair of fruits splitting at maturity to form a prominent scar on the nutlet and forming an acute angle with the thickened margin of the wing. *Deviacer* was initially presumed to be schizocarpic like *Acer*, but with the presumed attachment scar of each mericarp on the dorsal (rather than ventral) margin. However, no complete schizocarps have been recovered. *Deviacer* samaras have a very small and short rudder-like projection interpreted by Myers and Erwin to be a remnant style [6], (about 1.0–1.5mm long) arising from the nut opposite the pedicel attachment and protruding parallel to the long axis of the seed [5]. A similar projection occurs also in other asymmetrical samaras, e.g. *Phyllostylon* and *Cedrelospermum* (Ulmaceae) and *Securidaca* (Polygalaceae) [3].

*Deviacer* fossils have been reported from the Paleocene and late Eocene to early Oligocene of western North America [5,6,7,8,9,10,11,12], and in the Eocene of Europe [13]. The present fossils from the Oligocene sediment of the Ningming Basin in Guangxi, South China, are the first described *Deviacer* fossils from Asia, and also the lowest latitude *Deviacer* fossils. The Oligocene paleolatitude of the Ningming Basin is nearly the same as the present latitude [14]. These samara fossils provide significant evidence for the biogeographic history of the genus *Deviacer*.

## Material and Methods

**Fig 1:**
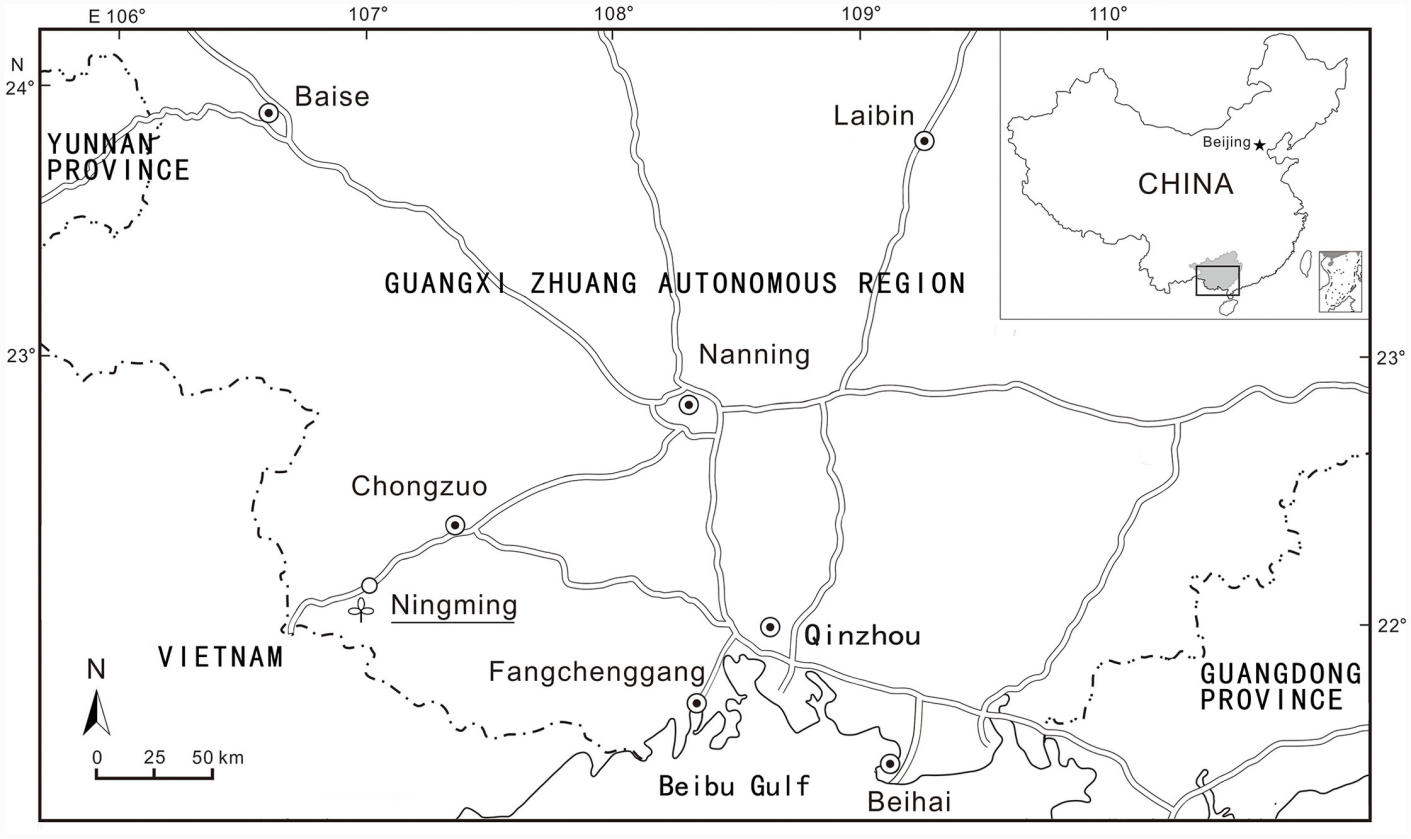
Sketched map showing the fossil locality (after [26]).

**Fig 2:**
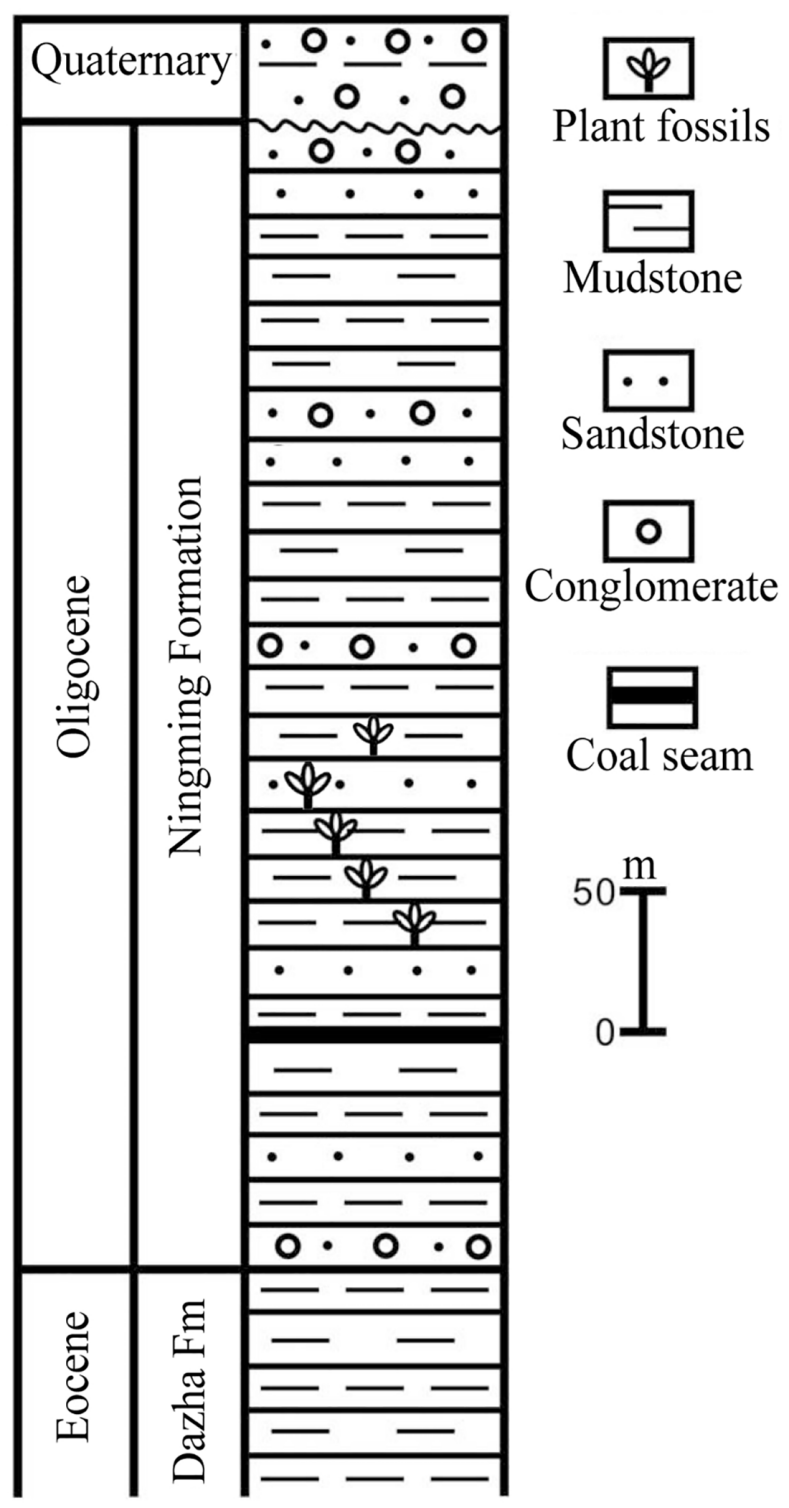
Generalized stratigraphic section of the Ningming Formation (after [22]).

The winged fruits were found together with various plant fossils from the Ningming flora in 2002. The flora is from a section of the Ningming Formation [15] at the Gaoling village in Ningming County, Guangxi Zhuang Autonomous Region, South China (22°07.690'N, 107°02.434'E) (Figs 1 and 2). The plant-bearing strata are a set of lacustrine deposits, mainly consisting of gray, dark gray mudstone intercalated with slightly yellow shaly siltstone and fine-grained sandstone, overlapping the Eocene Dazha Formation which is dominated by coarse quartz sandstone [1,15]. Because no volcanic material has been discovered at the locality, a radiometric dating was not available. The Paleogene of the Ningming Basin is divided into the Daza Formation and the Ningming Formation from the bottom upwards, referred to Eocene and Oligocene respectively [15]. The pollen assemblage of samples from the Ningming Formation is assigned an Oligocene in age [16].

The Ningming Formation yields abundant well-preserved plant and fish fossils [1,17,18,19,20]. Some plant megafossil species have been formally described and published [1,2,21,22,23,24,25,26,27]. Angiosperms dominate the flora, but gymnosperms and ferns are also present. The plant megafossils include Leguminosae, Sapindaceae (including Aceroideae), Juglandaceae, Lauraceae, Aceraceae, Betulaceae, Fagaceae, Hamamelidaceae, Moraceae, Ulmaceae, Simaroubaceae, Rutaceae, Araecaceae, Pinaceae, Taxodiaceae, Cupressaceae, Osmundaceae, and others.

The macromorphology and micromorphology of the fossils were photographed using a Nikon D800E camera with macro lens. The specimens described in this paper are housed at the Guangxi Museum of Natural History, Nanning, Guangxi Zhuang Autonomous Region, P.R. China. The wing venation nomenclature and terminology are after Wolfe & Tanai [4] and Manchester [5].

### Ethics Statement

All necessary permits were obtained for the described field studies and were granted by the local government of Ningming County of Guangxi.

## Description of Fossils

Family:? Polygalaceae Hoffmanns. and Link

Genus: *Deviacer* Manchester, emend. Myers and Erwin

*Deviacer guangxiensis* Chen et Manchester, sp. nov.

**Fig 3:**
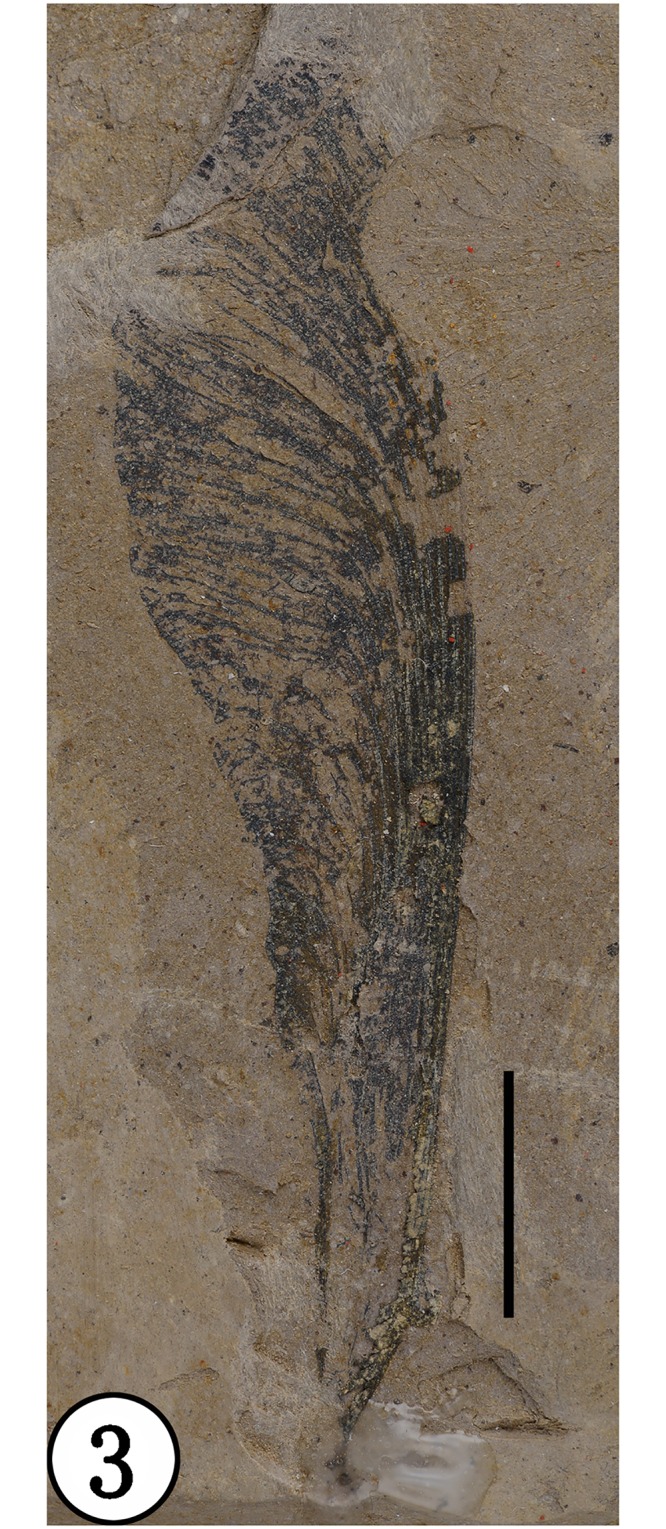
Holotype (NHMG030165), preserved at the Guangxi Museum of Natural History, China. Scale bar = 1 cm.

**Fig 4:**
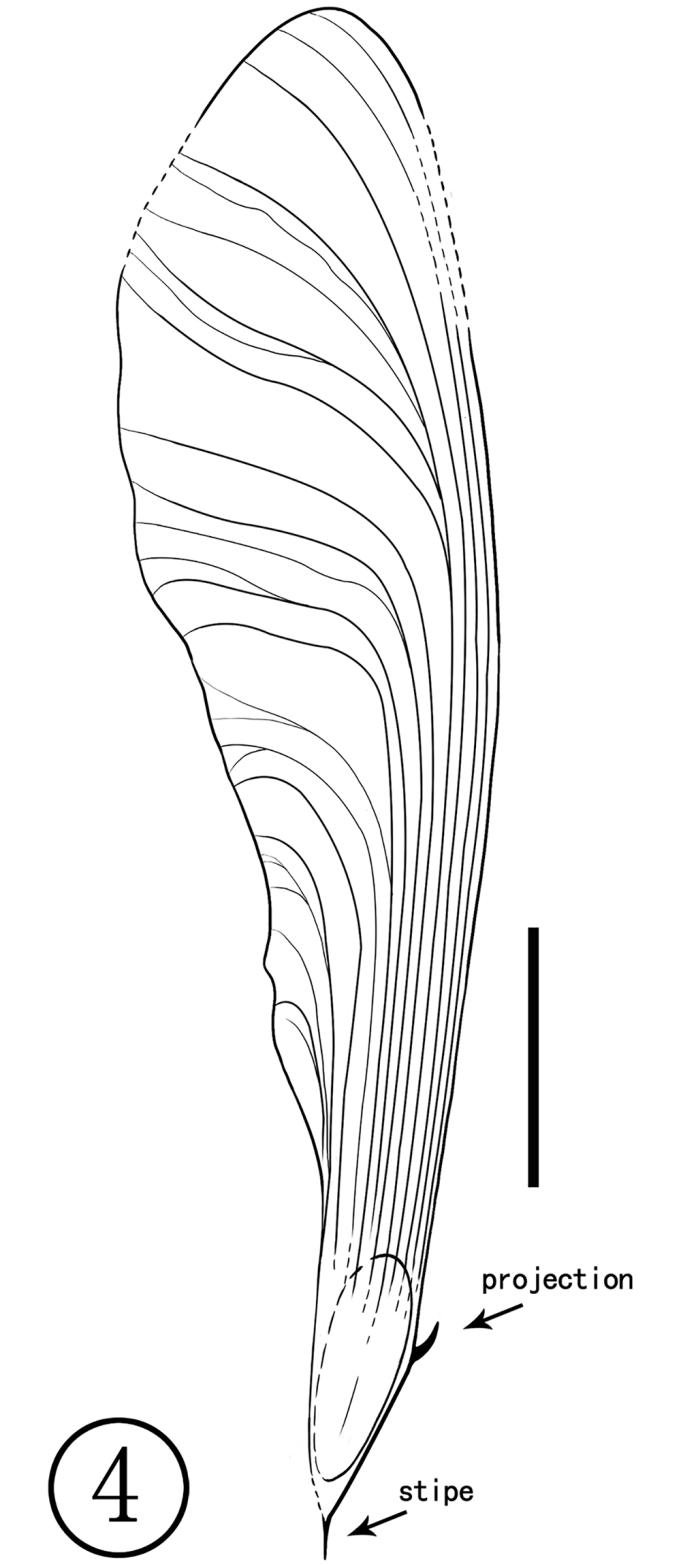
Line-drawing of Holotype (NHMG030165).

**Holotype**: NHMG030165 (Figs 3 and 4)

**Fig 5:**
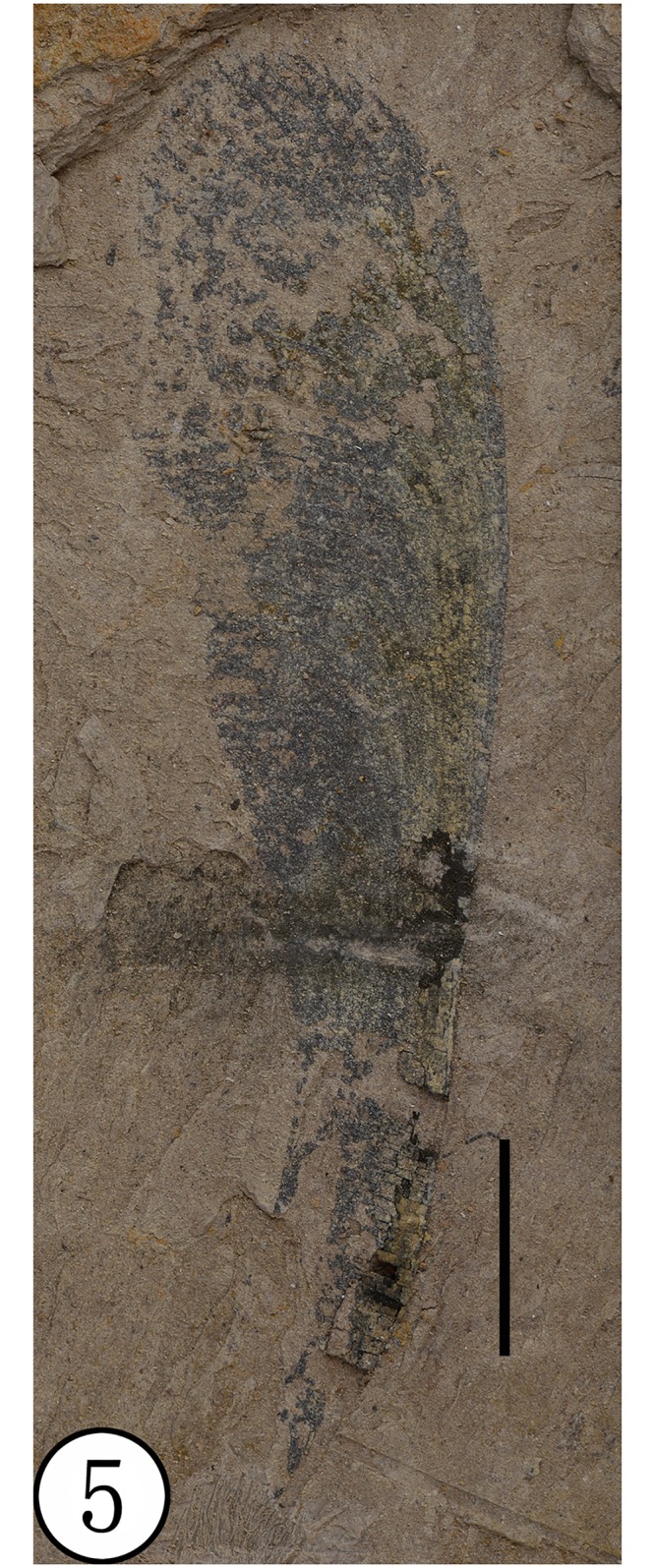
Paratype (NHMG030166), preserved at the Guangxi Museum of Natural History, China. Scale bar = 1 cm.

**Fig 6:**
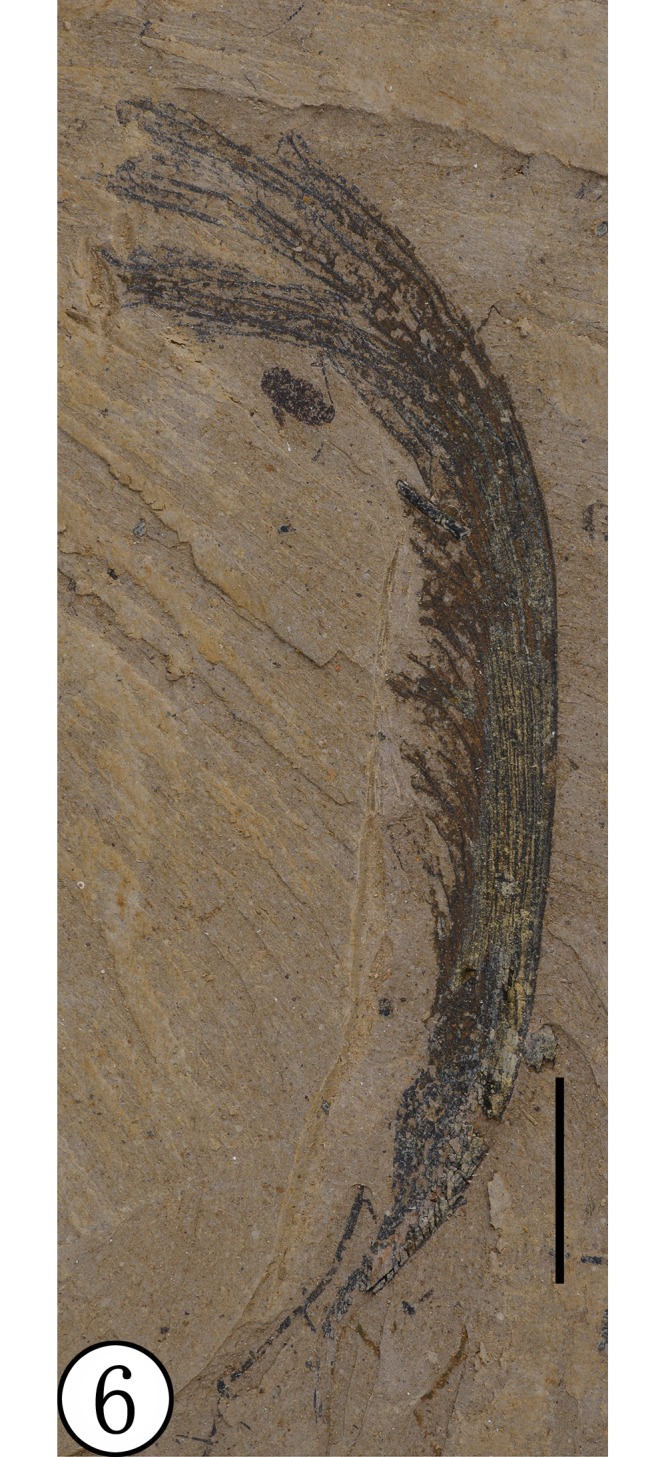
Paratype (NHMG030167), preserved at the Guangxi Museum of Natural History, China. Scale bar = 1 cm.

**Paratype**: NHMG030166, NHMG030167 (Figs 5 and 6)

**Repository**: Guangxi Museum of Natural History, China

**Type locality**: Ningming County, Guangxi, China (22°07.690'N, 107°02.434'E)

**Stratigraphic horizon**: Ningming Formation, Oligocene

**Etymology**: Species name referring to the occurrence of the fossils in Guangxi

**Diagnosis**: Samara asymmetric and large; nutlet fusiform, convex-rounded to straight on sharply angled and apparently stipitate proximally; proximal end of nutlet acute, forming a narrow angle of about 20–30°; wing dorsal margin relatively straight to slightly convex; ventral margin concave forming a broad and shallow sulcus proximal to the nut; in the wing body, venation subparallel, running to the dorsal margin for some distance then arching distally toward the ventral margin, with relatively few interreticulations; veins coalesced along the dorsal margin of the wing; stipe present, lacking basal perianth scar; pedicel not observed; a small projection arising from the nutlet opposite the attachment end of the fruit and proximal to the dorsal margin of the wing.

**Description**: Fruits are asymmetrical samaras that range from 59 to 65 mm long, up to 17 mm wide. The nutlet is fusiform, narrow, about 6–8 mm long, 3–4 mm wide, and 1.5 mm thick, with reticulate ribbing over the surface; some veins parallel on the surface of the nutlet, extending onto the wing. The dorsal margin of the wing is relatively straight to slightly convex, while the ventral margin is concave, proximally forming a broad and shallow sulcus adjacent to the nut. The wing apex is rounded. In the main part of the wing, venation is subparallel, running to the dorsal margin for some distance then arching distally toward the ventral margin, with relatively few interreticulations (anastomoses); veins very strong and coalesced along the dorsal margin of the wing. The stipe is at least 2mm long, without an obvious perianth scar at its base. A small projection about 1.5 mm long is present on the nut opposite the attachment end.

**Comparison**: *Deviacer wolfei* Manchester and *D*. *pidemarmanii* Myers & Erwin were the only two described species in the genus *Deviacer* [5,6]. Their samaras are smaller: *D*. *wolfei* about 20–34 mm long, *D*. *pidemarmanii* 25–41 mm long. The winged fruits described in this paper are much larger, about 59–65 mm long, and the veins of their wings are obviously stronger and more distinctly grooved. Compared with the other two species, this new species more closely resembles *D*. *pidemarmanii* in the very narrow shape of the nutlet. The nutlet narrows gradually into a stalk-like structure that appears at first to be a pedicel, but there is no indication of perianth attachment at junction of the nut and this stalk, so it is best considered a stipe [6]. The small projection on the nut proximal to the dorsal margin of the wing, is similar in position to the minute wing-like structure augmenting the large wing in *Securidaca* (Polygalaceae), but it is more stout and peg-like. In *Deviacer*, this might represent the remnant of a style [6]. Myers and Erwin [6] showed that some extant species of *Securidaca* have a stipitate nut similar to fossils of *Deviacer* and called attention to some fossils showing what appears to be the bulge of perianth scar at the base of the stipe, as in *Securidaca*. However, the present fossil has a very narrow and elongate stipe (Fig 5), unlike what we have seen in extant *Securidaca*. The stipe in extant *Securidaca paniculata* Rich. var. *lasiocarpa* Oort. is narrow and elongate, but relatively stout, and the fruit nutlet is round [6] rather than fusiform as in the present fossil. In addition, anatomical differences between similar permineralized fruits called *Paleosecuridaca* and extant *Securidaca* leads us to consider that the similarities in gross morphology among these fruits may be due to convergent evolution. Considering the unique characters of our specimens, we propose a new fossil species of *Deviacer* for the material from the Ningming Formation: *Deviacer guangxiensis* (see Table 1).

**Table 1:** Comparison among similar fruits.

Species	Nutlet shape	Nutlet average L/W	Samara size	Geographic location/age
*Paleosecuridaca curtisii*	ovate	1.3 : 1	25-36mm long, small	Eastern North America Paleocene
*Deviacer wolfei*	ovate	1.8 : 1	20-34mm long, small	Western North America Eocene
*Deviacer pidemarmanii*	fusiform	4 : 1	25-41mm long, small	Western North America Eocene-Oligocene
*Deviacer guangxiensis*	fusiform	3.8 : 1	59-65mm long, big	S.China Oligocene

L = length; W = width

## Discussion

Pigg, DeVore and Wojciechowski [12] established the genus *Paleosecuridaca* Pigg, DeVore & Wojciechowski, and the species *Paleosecuridaca curtisii* Pigg, DeVore & Wojciechowski, for permineralized asymmetric samaras from the late Paleocene of North America. They noted that those fossils greatly resemble *Deviacer* and may be the anatomically preserved equivalent. That species has somewhat larger fruits, 25 to 36mm long, than the previously reported samaras of *Deviacer wolfei*. It is probably equivalent to *Deviacer* as suggested by Pigg et al [12].

*Deviacer* has been reported from the Paleocene and Eocene or late Eocene—early Oligocene of North America [6], and the Early Eocene of Denmark (Fur Formation, H. Madsen and S. R. Manchester, unpublished data). The species described here shows that *Deviacer* inhabited the low latitude region in South China. Paleobotanical evidence suggests that this group of plants was widely distributed in the northern hemisphere during the Paleogene. It also provides evidence for the floristic exchange of this genus between western North America and eastern Asia before the Oligocene, via the Bering land bridge or via the North Atlantic land bridges. *Deviacer* fossils from North America and Denmark indicate that this plant seems to have inhabited warm temperate climatic conditions. Thus, we suggest that the climate at the time of deposition of the Ningming flora was somewhat cooler than at present, before the tropical monsoon climate prevails in this area at present. This is consistent with the climatic reconstruction based on physiognomic analysis of the Ningming flora [24].
